# Exogenous melatonin induces salt and drought stress tolerance in rice by promoting plant growth and defense system

**DOI:** 10.1038/s41598-024-51369-0

**Published:** 2024-01-12

**Authors:** Zakirullah Khan, Rahmatullah Jan, Saleem Asif, Muhammad Farooq, Yoon-Hee Jang, Eun-Gyeong Kim, Nari Kim, Kyung-Min Kim

**Affiliations:** 1 Department of Applied Biosciences, Graduate School, Kyungpook National University, Daegu, 41566 South Korea; 2 Coastal Agriculture Research Institute, Kyungpook National University, Daegu, 41566 South Korea

**Keywords:** Molecular biology, Plant sciences

## Abstract

Due to global climate change, crops are certainly confronted with a lot of abiotic and biotic stress factors during their growth that cause a serious threat to their development and overall productivity. Among different abiotic stresses, salt and drought are considered the most devastating stressors with serious impact on crop’s yield stability. Here, the current study aimed to elucidate how melatonin works in regulating plant biomass, oxidative stress, antioxidant defense system, as well as the expression of genes related to salt and drought stress in rice plants. Eight groups of rice plants (3 replicates, 5 plants each) underwent varied treatments: control, melatonin, salt, drought, salt + drought, salt + melatonin, drought + melatonin, and salt + drought + melatonin. Melatonin (100 µM) was alternately applied a week before stress exposure; salt stress received 100 mM NaCl every 3 days for 3 weeks, and drought stress involved 10% PEG. Young leaves were randomly sampled from each group. The results showed that melatonin treatment markedly reduces salt and drought stress damage by promoting root, shoot length, fresh and dry weight, increasing chlorophyll contents, and inhibiting excessive production of oxidative stress markers. Salt and drought stress significantly decreased the water balance, and damaged cell membrane by reducing relative water contents and increasing electrolyte leakage. However, melatonin treated rice plants showed high relative water contents and low electrolyte leakage. Under salt and drought stress conditions, exogenous application of melatonin boosted the expression level of salt and drought stress responsive genes like *OsSOS*, *OsNHX*, *OsHSF* and *OsDREB* in rice plants. Taken together, our results reveal that melatonin treatment significantly increases salt and drought tolerance of rice plants, by increasing plant biomass, suppressing ROS accumulation, elevating antioxidants defense efficiency, and up-regulating the expression of salt and drought stress responsive genes.

## Introduction

Rice (*Oryza sativa*) is one of the major staple food and widely cultivated throughout the world, and about half of the world population is depends upon rice^1^. As the population of world is increasing gradually, therefore the demand for the rice production is also increasing^2^. 21% of the calorific needs to the population of the world is provided by rice and south east Asia depends upon more than 76% for their calorific intake upon rice^3^. Continuous and rapid change in the climate may have serious impacts on the agricultural crops in the tropics and subtropics region by the end of this century^4^. Climate change is one of the major notable problem that alter the climate pattern, resulting droughts and extreme weather events^5^. Among the natural disasters, drought is one of the most dangerous disaster throughout the World, causing a significant ruin to ecosystems, agriculture and human societies^6^.

The major consequences of climate change are droughts which are tragic threat to water supplies, agriculture crops, food production and causing ecological disturbance and famine in the World^7^. Climate change and global warming are intensively affecting the regional and Worldwide hydrological cycle leading to the frequency of drought events^8^. Rice (*Oryza sativa*) as a paddy field agriculture crop which is significantly susceptible to droughts, and it is estimated that drought affected the World rice production nearly by 50%^9^. In Asia 75% of the total rice production comes from traditionally irrigated areas, which is facing the problem of droughts and water scarcity^10^.

At the same time salinity is an another environmental factor increasing in magnitude in the rice growing areas, due to the combine effects of high temperature, drought, sea level rising, and inferior agriculture practices^11^. In irrigated land, salt stress has been a serious threat to rice cultivation, and expected to be more than 20% in the near future, while it is estimated to reach 50% by 2050^12^. Salt stress reduce the rate of net carbon dioxide assimilation, growth of the leaf, enlargement of the leaf cell, accumulation of dry matter and relative growth^13^. The stress of salinity is dominated by sodium (Na^+^) and chloride (Cl^–^)^13^, negatively affecting rice growth and development due to creating ionic, osmotic and oxidative stress^13–15^. High level of salt in rice plant increase the toxicity level, leading to early leaf senescence and ultimately resulting the decrease in the photosynthetic leaf area^16,17^.

Melatonin (N-acetyl-5-methoxytryptamine) is common biological hormone that plays an important role in biological functions^18^, like circadian rhythms^19^, immunomodulation^20^, and oxidative stress reduction^21^. Hence during the past half century, it received great attention due to its anti-aging properties^22^. Recent studies showed that melatonin is present in a large number of vascular plants^22^, having an important role in germination^23^, lateral root formation, plant growth and defense against biotic and abiotic stresses^23,24^. Being a growth regulator, melatonin showed a great potential for enhancing plant drought resistance^25^. Melatonin has attracted the researcher to have an effective strategy to induce the crop tolerance against drought, salt, heavy metals, high temperature, low temperature, nutritional deficiencies and different types of diseases^26^. Melatonin can induce plant antioxidant system, help in plant photosynthesis improvement, enhance ion homeostasis and regulate plant hormone metabolism under the salt stress condition^27^. Pretreatment of rice with melatonin showed improvement in tolerance to salt stress by increasing plant’s fresh and dry weight and minimize plasma membrane damage^28^. Exogenous melatonin enhanced catalase (CAT), peroxidase (POD), superoxide dismutase (SOD) and ascorbate peroxidase (APX) activities in maize (*Zea mays*)^29^.

There is very limited information regarding the combined effects of salt and drought stress on rice plants, however salt and drought stress and its effects on rice were studied individually. A recent study on cotton (*Gossypium hirsutum*) shows that salt and drought combine stress cause significant reduction in plant growth, chlorophyll content and photosynthesis in cotton^30^. Combined stress reduced the activity of antioxidant enzymes which ultimately decreased the physiological performance of sunflower plants. Salinity and drought change the osmotic and ionic signal pathways in different crops^31^ *Salt Overly Sensitive* (*SOS*) pathway plays a key role in maintaining cellular ion homeostasis during salinity stress^32^. *OsNHX1* (Na^+^/H^+^* Exchanger*) is a transcription factor that regulates Na^+^ and K^+^ concentration of rice plants when exposed to NaCL and KCl stress^33^. Overexpression of *NHX1* in tobacco has been shown to confer salt tolerance^34^. Numerous transcription factors such as *DREB,* (*Dehydration-Responsive Element-Binding*) *ABRE* (*ABA-responsive element*) and *ERF* (*Ethylene Responsive Factor*) have been identified playing an important role in transcriptional regulation under drought stress^35,36^. Overexpression of *HSF2* (*Heat Shock Transcription Factor*) improved drought tolerance at the seedling stage in Arabidopsos^37^. *HSFA3* and *HSFA1b* take part in different signaling pathways to enhance the plant’s tolerance to drought stress while, the expression of *HSFA3* in response to drought stress is dependent upon the expression of *DREB2A*^38^.

Our study hypothesized that induction of melatonin enhances tolerance to individual as well as combined drought and salt stress in the rice plant. The aim of our study is to evaluate the role of melatonin in rice plants in response to drought and salt stress individually and when they are combined. We focused on the effects of melatonin on morphological parameters, antioxidants and transcriptional regulation of salt and drought responsive genes in response to salt and drought combined stress.

## Materials and methods

### Plant material and growth conditions

Ilmi rice cultivar (*Oryza sativa* L.) seeds were used in this experiment, provided by Plant Molecular Breeding Laboratory, Kyungpook National University, Korea^39^. Rice seeds were sterilized with fungicides for a night followed by washing with double distilled water three times. Then, the rice seeds were kept in water for 4 days in an incubator in the dark condition at 32 °C, changing the water after each 24 h as previously reported by Ref.^39^. After germinating for three days, the seeds were transplanted into plastic pots having I L capacity filled with specialized soil mix (Doobaena plus) consisting of cocopeat (27%), peat moss (10%), vermiculite (34%), Masato (10%), diatomite (13%), bara mesh (5.5%), fertilizer (0.48%), and humectant (0.2%), provided by Nongkyung Co. Ltd, Korea, to foster their growth. The seeds were grown for three weeks in greenhouse for further experiments.

### Experimental design

In this experiment, a total of eight groups of rice plants were involved. Each of these groups had three replicates, and within each replicate, there were five plants. The experimental groups were control plants (C), melatonin treated plants (M), salt treated plants (S), drought treated plants (D), salt** + **drought treated (S + D), salt + melatonin treated plants (S + M), drought + melatonin treated plants (D + M) and salt + drought + melatonin treated (S + D + M). Water was applied on daily basis. Before 1 week the salt and drought stress exposure plants were treated with 100 µM of melatonin at alternative days as described by Ref.^40^, and our preliminary screening. For the salt stress, plants were treated with 100 mM of NaCl at three days interval for 3 weeks^41^. For drought stress 10% PEG polyethylene glycol 6000 (PEG 6000; a product of Sigma-Aldrich, Seoul, Korea) was applied, according to method used by Ref.^42^. The experiment involved the random selection of the young fully expanded leaves from each experimental group.

### Analysis of morphological parameters and biomass

After 35 days of plant growth, shoot length, root length, height of shoot, and fresh weight (FW) of the rice plants were measured. For the determination of the dry weight (DW) of seedlings, the roots and shoots were dried by oven at 200 ℃ for 30 min and maintained at 60 ℃ for 48 h to obtain DW. The fresh leaves were put into a petri dish filled with distilled water and the petri dishes were placed in a dark environment for 4 h, and then their turgid weight (TW) was recorded, after that the leaves were oven dried to obtain DW. Finally, relative water content (RWC) was quantified using formula: $RW=\frac{FW-DW}{TW-DW}\times 100$^43^.

### Chlorophyll contents

Chlorophyll contents were measured after 1 week of stress exposure by using portable chlorophyll meter (SPAD 502, Konica Minolta, Japan). The second last fully mature leaf was selected for chlorophyll measurement and the reading was taken from leaf base, middle and near the leaf tip. Five leaves were measured from each treatment group for chlorophyll contents and the average value was taken as SPAD value as mentioned previously^44^.

### Electrolyte leakage

For determination of electrolyte leakage, fresh leaves samples were cut into 5 mm and placed in test tubes containing 10 mL deionized water. The tubes were covered with plastic caps and placed in a water bath maintained at the constant temperature of 32 °C. The initial electrical conductivity (ECI) was measured after 2 h by electrical conductivity meter (CM-115, Kyoto Electronics, Kyoto, Japan). Then the samples were autoclaved at 121 °C for 20 min to release all electrolytes and kill the tissues. For measurement of final electrical conductivity (EC2), samples were cooled to 25 °C. Electrolyte leakage (EL) was find out using formula: $EL=\frac{EC1}{EC2}\times 100$.

### Determination of H_2_O_2_ and MDA contents

H_2_O_2_ contents were measured using previously described method^45^. Briefly, fresh leaves of 0.1 g were ground in liquid nitrogen, extracted in 5 mL of 0.1% TCA and centrifuged at 12,000×*g* for 15 min. Supernatant of 0.5 mL was taken, potassium iodide 1 mL (1 mM) and potassium phosphate buffer (pH 7.0) 0.5 mL of (10 mM) were added, and the absorbance was measured at 390 nm. Using the extinction coefficient (ɛ) 0.28 mM cm^−1^, H_2_O_2_ content was estimated and expressed as µmol g^−1^ of FW. MDA contents were determined as previously described by Ref.^46^. In brief, fresh plant leaves of 0.1 g were ground in 10 mL of TCA 5% and centrifuged at 4000×*g* for 10 min at 4 °C. The supernatant was taken in 4 mL of TBA, incubated at 90 °C for 25 min and then cooled down at 4 °C. The supernatant was read at of 532 and 600 nm. The MDA content was measured as µmol g^−1^ of FW.

### Determination of antioxidative activities

Catalase activity was find out by the method of Ref.^47^ briefly, crude enzymes was treated with 0.5 mL of 0.2 mM H_2_O_2_ using sodium phosphate buffer with 7 pH. The activity of catalase was determined by the decrease in the absorbance of H_2_O_2_ at 240 nm, and CAT one unit was defined as micromoles of hydrogen peroxide (H_2_O_2_) decomposed per minute per milligram of protein. Reduced glutathione contents were determined using the protocol of Ref.^48^, in brief, fresh leaves were ground in liquid nitrogen, 2 mL 10% (v/v) trichloroacetic acid was added and centrifuged at 4 °C for 13 min at 10,000×*g*. The supernatant was combined with 3 mL of 150 mM NaH_2_PO_4_ (pH 7.4). Then nitrobenzoic acid (75.3 mg of DTNB dissolved in 30 mL of 100 Mm sodium phosphate buffer, pH 6.8) was added, followed by incubation for 5 min at 30 °C. At 412 nm the absorbance of the samples was measured, with reference to standard curve, reduced glutathione concentration was calculated and expressed as (nmol g^−1^ FW). All the experiments were performed three times.

### RNA isolation and qRT-PCR

To determine expression level of *OsHSF, OsDREB, OsNHX* and *OsSOS* gene, from each group rice leaves were collected randomly at 0, 12, 24 and 48 h after the plants were exposed to stress. Using RNeasy Plant Mini Kits (50) Qiagen, RNA was extracted, cDNA was synthesized using qPCRBIO kits, while using qPCRBIO SYBR Green kits, qRT-PCR was performed. Primer sequence and accession number of each gene are shown in Table 1. 20 µL of reaction was started using 10 µL SYBR green, 7 µL ddH2O, 1 µL template DNA, and 1 µL of each primer. The reaction was incubated at 95 °C for 2 min, followed by thirty-five cycles at 94 °C for 10 s, and 60 °C and 72 °C for 10 and 40 s, respectively. Each reaction was performed three time using actin as an internal reference gene.

**Table 1:** Primers and accession numbers of selected genes designed by NCBI for qRT-PCR.

Gene	Forward primers	Reverse primers	Accession no
*OsActin*	CTGCGGGTATCCATGAGACT	GGAGCAAGGCAGTGATCTTC	X16280.1
*OsHSF*	GCGAGAGAAGCTCAGCTAGG	CCCAGACGTAGAATCCGGTG	AK101182
*OsDREB*	AGGAGGGAGAAATCTGGCAC	CGCACTGAAAAGTGTGGACA	AK062422
*OsNHX*	GCGGTGCATTTTGCTCTCAA	CCTGCTTCAGATCAGGGTGG	AK104336
*OsSOS*	TCGCAGACAGGGTGTTTGAT	CGCTTTTGGGTGGAACACAC	AK101368

### Statistical analysis

**Figure 1:**
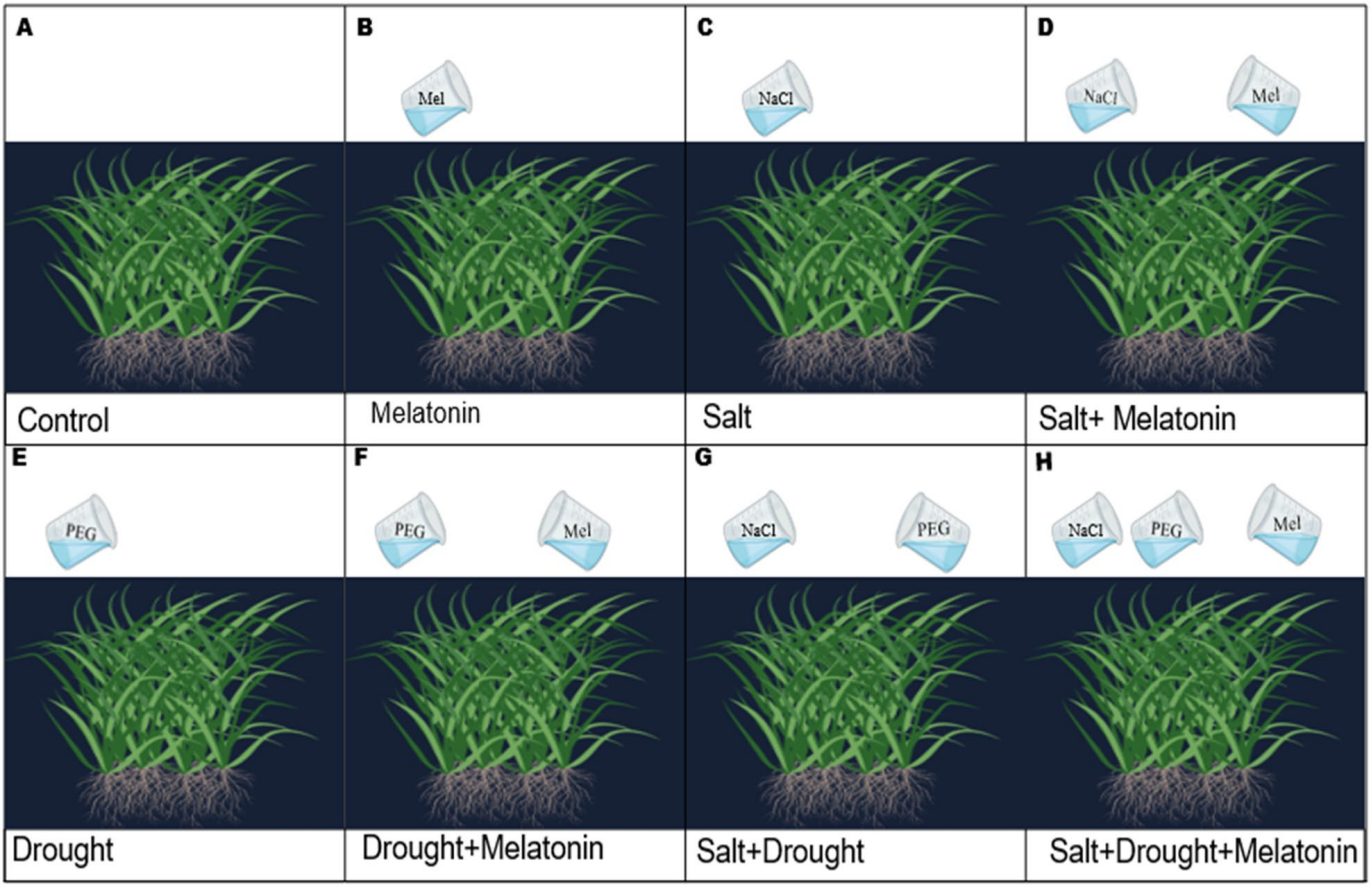
Experimental design of the experiment, indicating the eight experimental boxes and application of drought and salt stress. Box (**A**) shows control plants. Box (**B**) shows plants treated with melatonin only. Box (**C**) shows the plants exposed to salt stress. Box (**D**) shows melatonin treated plants exposed to salt stress. Box (**E**) shows plants exposed to drought stress. Box (**F**) shows melatonin treated plants exposed to drought stress. Box (**G**) shows plants exposed to salt and drought combined stress. Box (**H**) melatonin treated plants exposed to salt and drought combined stress.

All experiments were performed three time. Data were analyzed using two-way ANOVA with Bonferroni post hoc tests (*shows p < 0.05 and **shows p < 0.01 significant difference). For the comparison of the mean values of different treatments completely randomized design was used. Data were graphically presented, and statistical analyses were calculated using the GraphPad Prism software (version 5.01, GraphPad, San Diego, CA, USA) and Statistical Analysis System (SAS 64 bit, developed by North Carolina State University, Raleigh, NC, USA) (Fig. 1).

## Results

### Plant growth under salt/drought stress and effects of melatonin

**Figure 2:**
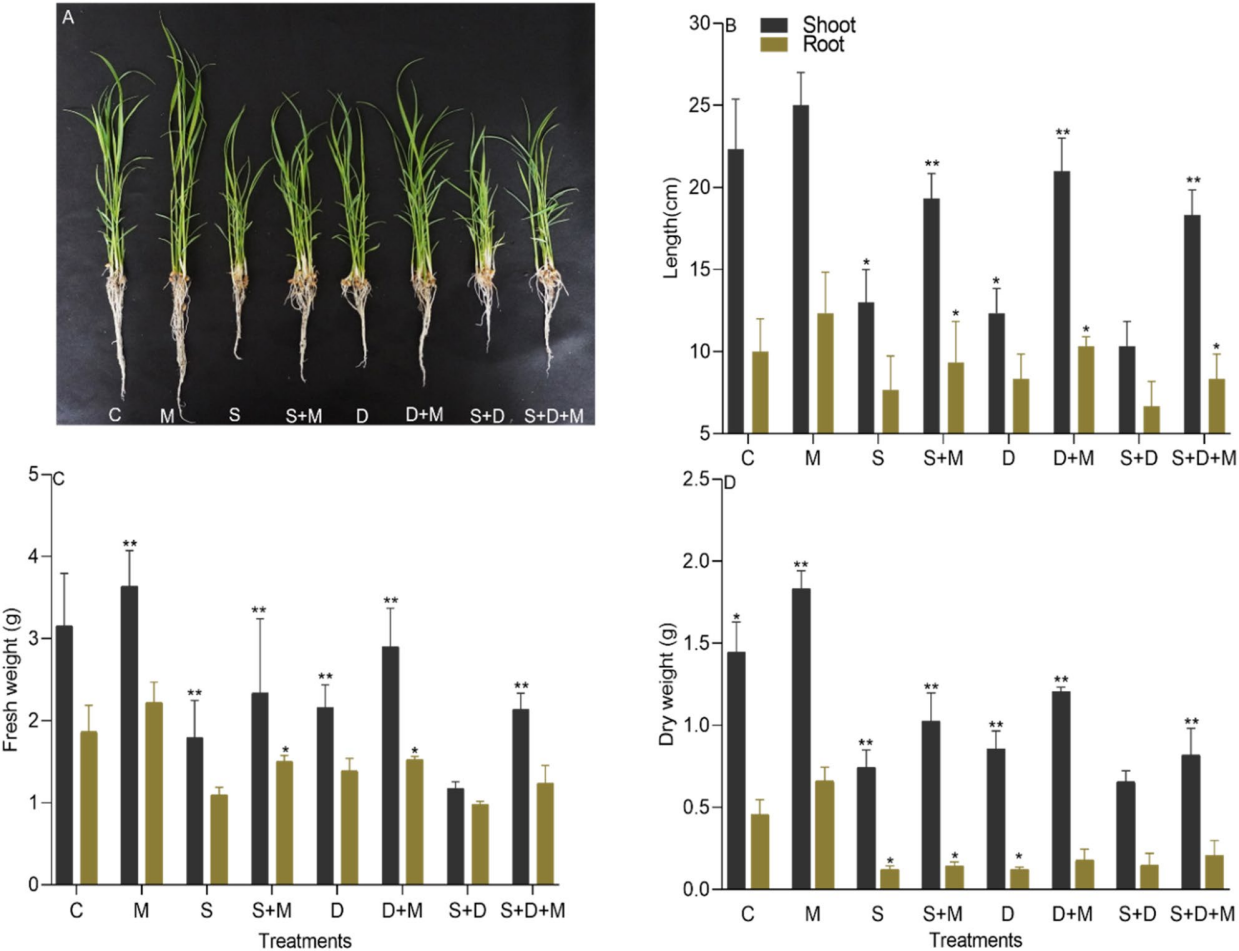
Evaluation of growth parameters in rice plants under salt and drought stress. Figures show that melatonin increased the root shoot length and plant biomass in salt and drought stress individually as well as in combined. (**A**) Shows the salt and drought stress damage. (**B**) Shoot length, (**C**) root length, (**D**) fresh and dry weight. Data were analyzed in three independent biological replicates (± standard deviation, SD), and the means were compared using Dunnett tests. *Indicates p and < 0.05, **indicates p < 0.01. Whereas C is control, M is melatonin, S is salt, (S + M) is salt + melatonin, D is drought, (D + M) is drought + melatonin, (S + D) is salt + drought and (S + D + M) is salt + drought + melatonin.

In this experiment, we evaluated various growth parameters of rice plants in response to salt and drought combined and individual stress as shown in (Fig. 2A). Both salt and drought stress significantly reduced plant shoot length by 23% in (S), 20% in (D), and 32% in (S + D), and root length by 25% (S), 18% (D), and 32% (S + D) compared to (C). However, melatonin treated plants (M) resulted in a 22% increase in plant height compared to (S + M), 16% to (D + M), and 28% to (S + D + M) and increase in root length by 29% to (S + M), 22% to (D + M), and 33% to (S + D + M) respectively. Additionally, the application of exogenous melatonin notably raised plant shoot length by 53% in (S + M), 75% in (D + M), and 80% in (S + D + M), respectively. Likewise, melatonin showed an increase in plant root length by 21% in (S + M), 24% in (D + M), and 24% in (S + D + M) as compared to their respective stress (S), (D), and (S + D) conditions (Fig. 2B). Salt and drought stress significantly declined shoot fresh weight (43%, 31%, and 59%), shoot dry weight (48%, 40%, and 54%), root fresh weight (39%, 27%, and 25%), and root dry weight (60%, 53%, and 73%) in (S), (D), and (S + D) when compared to (C). Plants treated exclusively with melatonin (M) exhibited enhanced shoot fresh weight (36%, 24%, and 69%), shoot dry weight (31%, 36%, and 42%), root fresh weight (27%, 33%, and 45%), and root dry weight (23%, 31%, and 39%) in comparison to (S + M), (D + M), and (S + D + M). Similarly exogenous application of melatonin significantly induced increases in both fresh and dry weights of the shoot and root. Shoot fresh weight saw increments of 30.16% in (S + M), 34.25% in (D + M), and 82.05% in (S + D + M), while root fresh weight exhibited increases of 37.12% in (S), 9.35% in (D), and 26.80% in (S + D) compared to (S), (D), and (S + D) (Fig. 2C). Moreover, the dry weight of the shoot was enhanced by 37.83% in (S + M), 14.35% in (D + M), and 26.15% in (S + D + M), while the dry weight of the root experienced increments of 16.10% in (S + M), 41.30% in (D + M), and 42.85% in (S + D + M) as compared to (S), (D), and (S + D) (Fig. 2D).

### Effects of exogenous melatonin on chlorophyll contents

**Figure 3:**
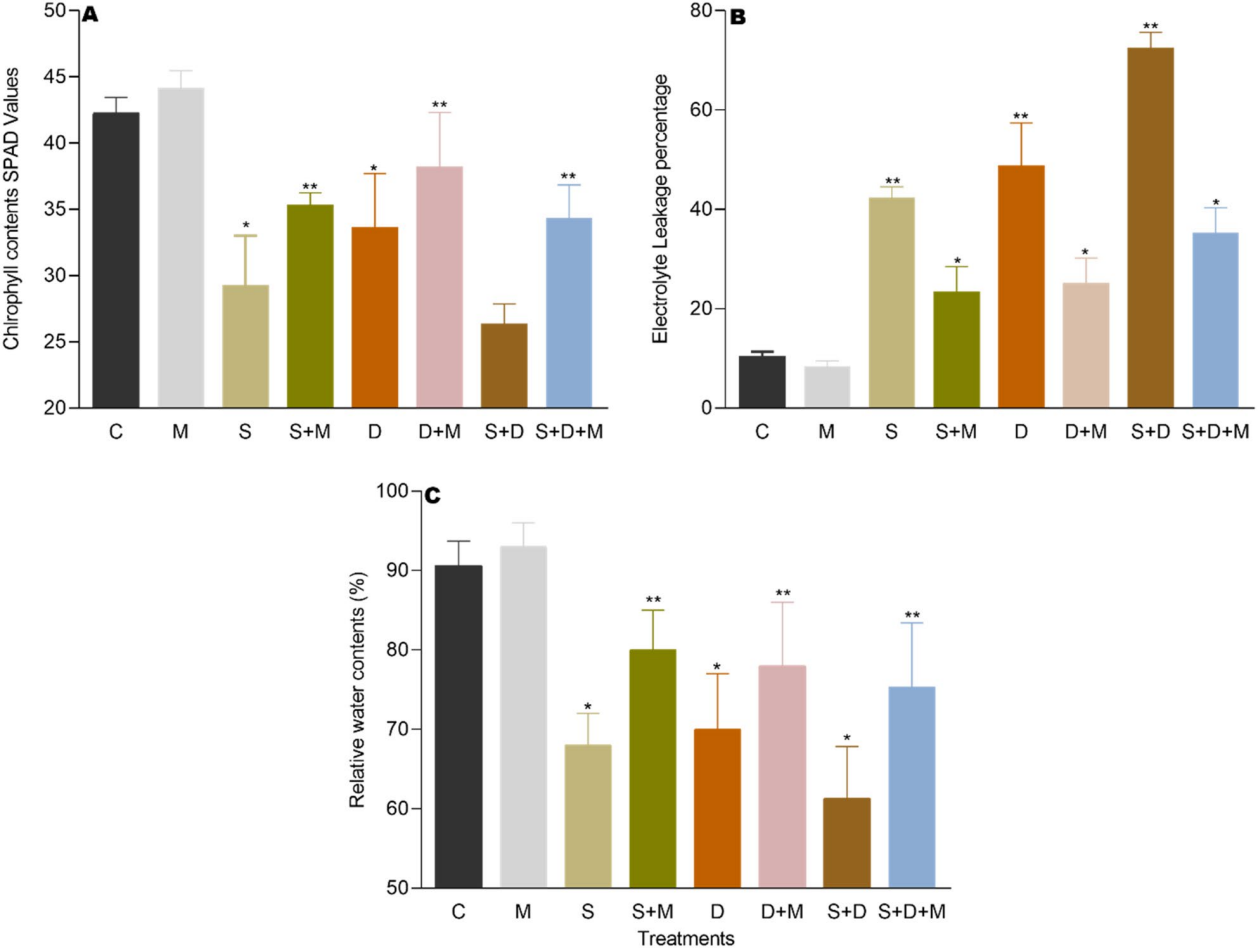
Melatonin application reduces salt and drought stress effects on chlorophyll contents. (**A**) Shows chlorophyll contents SPAD values. (**B**) Electrolyte leakage and (**C**) relative water contents which are regulated by exogenous treatment of melatonin. Data were analyzed in three independent biological replicates (± standard deviation, SD), and the means were compared using Dunnett tests. *Indicates p < 0.05 and **indicates p < 0.01.

Salt and drought stress resulted in a significant decrease in chlorophyll contents by 31%, 22%, and 38% in (S), (D), and (S + D) compared to the control (C). However, 29%, 16%, and 27% of increment was observed in melatonin treated plants (M) as compared to (S + M), (D + M), and (S + D + M). Likewise, melatonin treatment led to a notable increase in chlorophyll contents, with increments of 20.33% in (S + M), 13.20% in (D + M), and 30.23% in (S + D + M) compared to (S), (D), and (S + D) conditions (Fig. 3A).

### Effects of exogenous melatonin on electrolyte leakage

Membrane permeability, as indicated by electrolyte leakage, is notably influenced by both salt and drought stress by 220% in (S), 190% in (D), and 410% in (S + D) as compared to (C). Although Melatonin treatment (M) markedly decreased electrolyte leakage by 65%, 68%, and 77% compared to (S + M), (D + M), and (S + D + M), respectively. Furthermore, exogenous melatonin treatment significantly decreased the electrolyte leakage by 44.51% in (S + M), 48.57% in (D + M) and 51.35% in (S + D + M) respectively as compared to (S), (D), and (S + D) (Fig. 3B).

### Effects of exogenous melatonin on relative water contents

As a measure of plant water status, Relative Water Content (RWC) not only provides insights into the hydration level of a plant but also serves as a reflection of its metabolic activity^49^. RWC is closely related to physiological function of plants, and it also indicates the ability of plant to sustain its water contents and wilting degree of leaves^50^. A significant reduction of 24% under salt stress (S), 22% under drought stress (D), and 32% under combined salt and drought stress (S + D) was recorded compared to the control (C). While RLW contents were 16% 19% and 24% higher in melatonin-treated plants (M) as compared with (S + M), (D + M), and (S + D + M). Similarly, melatonin-treated plants exhibited a significant increase in relative water contents, registering a rise of 17.76% in (S + M), 11.92% in (D + M), and 22.82% in (S + D + M) when compared to (S), (D), and (S + D) conditions (Fig. 3C).

### Effects of exogenous melatonin on H_2_O_2_ and MDA contents

**Figure 4:**
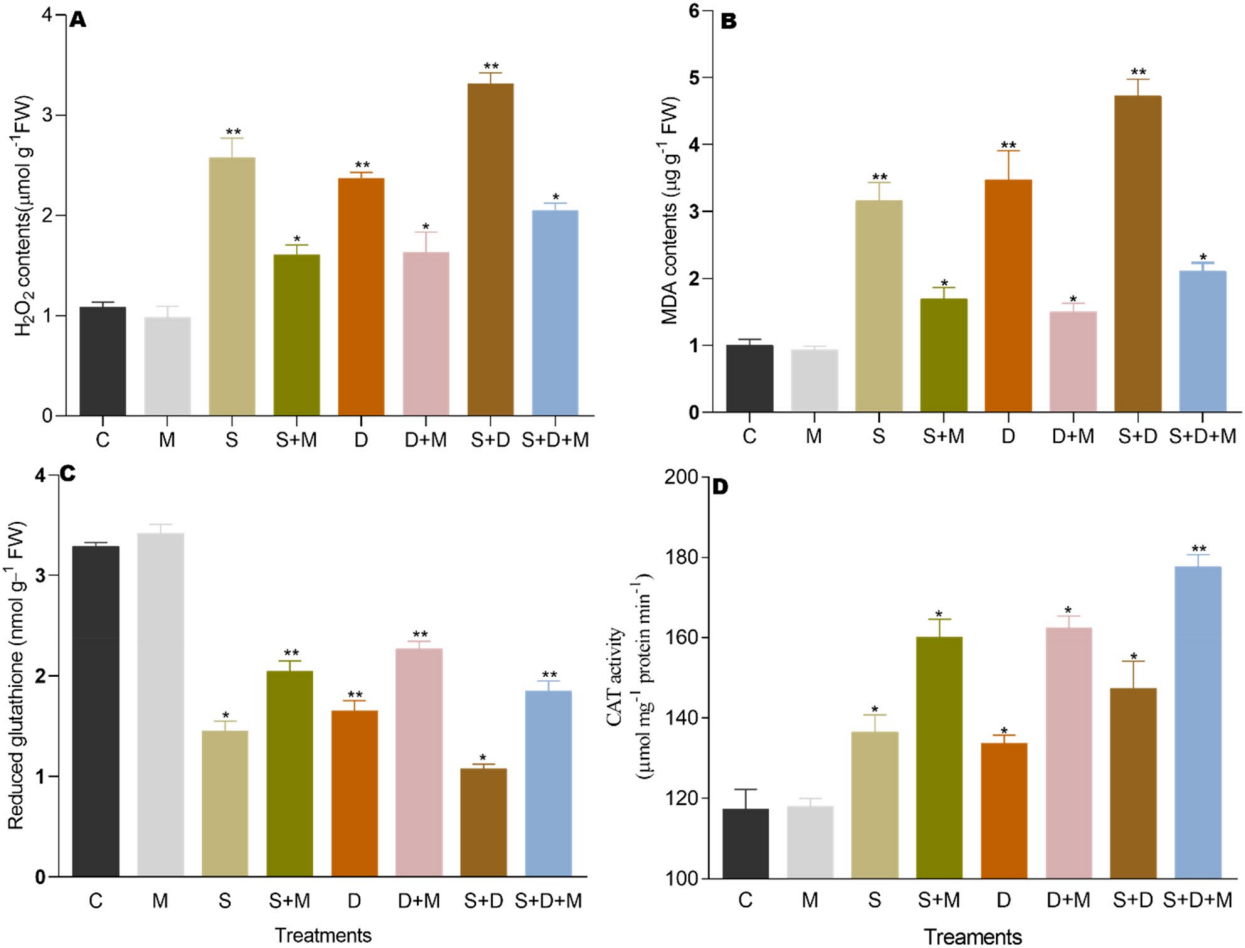
Melatonin application alleviates salt and drought stress by scavenging ROS accumulation. (**A,B**) Shows H_2_O_2_ and MDA contents. Melatonin also regulates the activities of antioxidants. (**C**) Shows the activities of Glutathione reductase (GR) and (**D**) shows the activities of catalase (CAT). Data was analyzed in three independent biological replicates (± standard deviation, SD), and the means were compared using Bonferroni post hoc tests. *Indicates p < 0.05 and **indicates p < 0.01.

Hydrogen peroxide serves as an indicator of the reactive oxygen species (ROS) scavenging capacity in plants under various stresses, and it is generated as a byproduct of cellular metabolism. The results show that H_2_O_2_ showed a substantial increase of 131% under salt stress (S), 112% under drought stress (D), and 206% under combined salt and drought stress (S + D) compared to the control (C). Melatonin-treated plants (M) exhibited a reduction in H_2_O_2_ contents by 43%, 45%, and 56% compared to the levels observed in plants under salt stress (S), drought stress (D), and combined salt and drought stress (S + D), respectively. Furthermore, exogenous application of melatonin demonstrated a substantial reduction in H_2_O_2_ accumulation, showing decreases by 37% in (S + M), 31% in (D + M), and 39% in (S + D + M) compared to (S), (D), and (S + D) (Fig. 4A).

Salt and drought stress demonstrated a notable impact on Malondialdehyde (MDA) contents, elevating the levels by 210%, 250%, and 370% in salt-stressed (S), drought-stressed (D), and combined salt and drought-stressed (S + D) plants, respectively, compared to the control (C). In contrast, melatonin-treated plants (M) exhibited a significant reduction in MDA levels by 45%, 38%, and 55% compared to plants under salt and melatonin treatment (S + M), drought and melatonin treatment (D + M), and combined salt, drought, and melatonin treatment (S + D + M), respectively. Similarly, MDA contents were significantly decreased by melatonin treatment, recording reductions of 46% in (S + M), 56% in (D + M), and 55% in (S + D + M) compared to plants under salt stress (S), drought stress (D), and combined salt and drought stress (S + D) conditions, respectively (Fig. 4B).

### Melatonin reduce oxidative stress via regulation of GR and CAT

Melatonin provides protection to plants from oxidative damage through the activation of antioxidants. In this study, the effects of salt and drought stress on the antioxidant activities of glutathione (GSH) and catalase (CAT) in rice plants were investigated, with and without melatonin treatment. The results revealed a significant reduction in glutathione (GSH) by 56%, 53%, and 66% in salt-stressed (S), drought-stressed (D), and combined salt and drought-stressed (S + D) plants compared to the control (C). In contrast, melatonin-treated plants (M) exhibited a rise in glutathione (GSH) levels by 70%, 47%, and 80% compared to plants under salt and melatonin treatment (S + M), drought and melatonin treatment (D + M), and combined salt, drought, and melatonin treatment (S + D + M), respectively. Similarly, melatonin increased glutathione (GSH) activities by 40% in (S + M), 36% in (D + M), and 72% in (S + D + M) compared to plants under salt stress (S), drought stress (D), and combined salt and drought stress (S + D) (Fig. 4C).

The catalase (CAT) activity showed a gradual increase due to salt and drought stress; however, melatonin treatment accelerated its activity. The catalase (CAT) activity increased by 16% under salt stress (S), 14% under drought stress (D), and 25% under combined salt and drought stress (S + D) compared to the control (C). Catalase (CAT) was also recorded higher by 26%, 27%, and 34% in salt and melatonin-treated (S + M), drought and melatonin-treated (D + M), and combined salt, drought, and melatonin-treated (S + D + M) conditions compared to melatonin-treated plants (M). Exogenous melatonin treatment significantly increased catalase (CAT) activity by 17.64% in (S), 21.80% in (D), and 20.40% in (S + D) respectively (Fig. 4D).

### Melatonin regulates the salt and drought stress responsive genes

**Figure 5:**
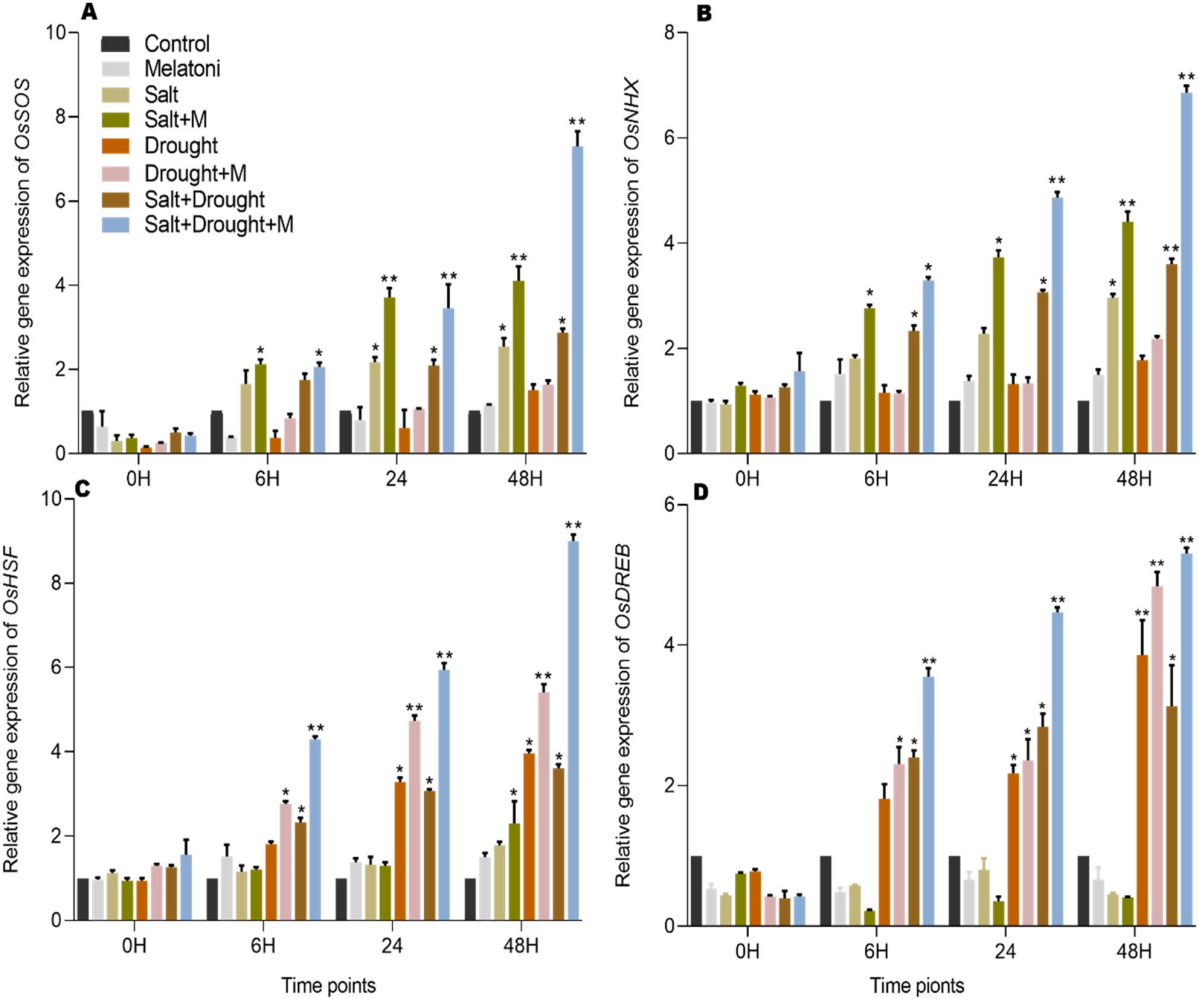
Melatonin reduces salt and drougth stress via regulation of drought and salt stress responsive genes. (**A,B**) Show the relative expression of *OsSOS* and *OsNHX* salt stress responsive gene while, (**C,D**) show the relative expresssion of *OsHSF* and *OsDREB* drought responsive genes in rice plant respectively. Data were analyzed in three independent biological replicates (± standard deviation, SD), and the means were compared using Bonferroni post hoc tests. *Indicates p < 0.05 and **indicates p < 0.01.

The combined effects of salt and drought stress exert a considerable influence on the expression of genes associated with these stressors. The expression of *OsSOS* was observed as 60%, 110%, and 150% under salt stress (S), and 70%, 109%, and 190% under combined salt and drought stress (S + D) at 6, 24, and 48 h following the application of stress, in comparison to control plants (C). Additionally, *OsSOS* exhibited a significant increase by 155%, 311%, and 340% in (S + M), and 100%, 240%, and 300% in (S + D + M) after 6, 24, and 48 h, respectively, following stress application, in comparison to the expression in melatonin-treated plants (M). Melatonin demonstrated a significant up-regulation in the expression of *OsSOS* by 43%, 63%, and 76% in (S + M) after 6, 24, and 48 h following stress application. Additionally, in (S + D + M), the expression was increased by 35%, 104%, and 151% after 6, 24, and 48 h following stress application, respectively, when compared to (S) and (S + D) (Fig. 5A). Similarly, salt and drought stress induced the expression of *OsNHX* by 50%, 70%, and 170% in (S), and 130%, 221%, and 350% in S + D after 6, 24, and 48 h of stress, respectively, compared to control (C) plants. *OsNHX* displayed a notable increase of 80%, 140%, and 193% in (S + M), and 113%, 220%, and 333% in (S + D + M) after 6, 24, and 48 h, respectively, subsequent to stress exposure, in contrast to the expression observed in plants treated solely with melatonin (M) Melatonin up-regulated the expression of OsNHX by 50.25%, 71.54%, and 51.56% in (Salt + Melatonin) after 6, 24, and 48 h, and 53.25%, 75.64%, and 88.69% in (S + D + M) after 6, 24, and 48 h following stress application, respectively, as compared to (S) and (S + D) (Fig. 5B).

Similarly, the treatment of exogenous melatonin also induced the expression of drought responsive genes *OsHSF* and *OsDREB*. *OsHSF* exhibited a significant increment of 80%, 220%, and 269% in drought-stressed (D), and 133%, 206%, and 260% in salt and drought-stressed (S + D) after 6, 12, and 24 h of stress, respectively, compared to (C). OsHSF exhibited a substantial increase of 80%, 188%, and 217% in (S + M), and 147%, 248%, and 423% in (S + D + M) after 6, 24, and 48 h, respectively, following stress exposure, compared to the expression observed in plants treated solely with melatonin (M). Melatonin treatment increased the expression of *OsHSF* by 50%, 47%, and 38% in (D + M) after 6, 24 and 48 h and 82%, 95% and 147% in (S + D + M) after 6, 24 and 48 h of applying the stress respectively as compared to (D) and (S + D) (Fig. 5C). Salt and drought stress resulted in a significant increase in *OsDREB* expression, with increments of 80%, 117%, and 285% in drought-stressed (D), and 140%, 184%, and 212% in salt and drought-stressed (S + D) after 6, 12, and 24 h of stress, respectively, compared to control (C). OsDREB exhibited a significant increase of 130%, 135%, and 380% in (S + M), and 191%, 266%, and 342% in (S + D + M) after 6, 24, and 48 h, respectively, following stress exposure, compared to the expression observed in plants treated solely with melatonin (M). Similarly, melatonin significantly increased the expression of *OsDRED* by 27%, 9% and 28.% in (D + M) after 6, 24 and 48 h and 48%, 78% and 98% in (S + D + M) after 6, 24 and 48 h respectively as compared to (D) and (S + D) (Fig. 5D).

## Discussion

In this study, both salt and drought stress, either independently or in combination, resulted in a substantial depletion in rice growth (Fig. 2). The inhibition of new leaf growth and the development of the root system due to drought and salt stress are widely acknowledged factors contributing to the reduction in biomass accumulation^41,51^. Salinity and drought stress significantly reduce seed germination rates, shoot, and root length, as well as the overall biomass of rice seedlings, resulting in hindering plant growth^52,53^. This study confirms that both salinity and drought stress severely restricted the growth and development of rice, as shown in (Fig. 2A,B). And these stressors significantly reduced both above-ground fresh weight and dry weight, as depicted in (Fig. 2C,D). Moreover, the inhibitory effect on plant growth and biomass was more pronounced under salt stress compared to drought stress. Furthermore, chlorophyll plays essential roles in plant growth, development, and the synthesis of photosynthetic products. Salt stress hinders chlorophyll synthesis, directly impacting photosynthesis, retarding plant growth, and diminishing yield^54,55^. In this experiment, during salt and drought stress, chlorophyll contents were significantly reduced (Fig. 3A). This may be due to rise in the level of Na^+^, MDA, and H_2_O_2_, disrupting chloroplast membrane stability and causing degradation of the protein-pigment-lipid complex^56^. Exogenous application of melatonin reversed the downward trend and promoted plant growth, biomass, and chlorophyll contents (Fig. 3C,D). These findings align with prior research^57^, suggesting the potential impact of melatonin on enzymes contributes to the enhancement of chlorophyll level^58^, thereby promoting plant growth and development.

The findings from this study indicate that elevated salt and drought stress levels led to a decrease in RLWC (Relative Leaf Water Content). This decline in RLWC may have contributed to a reduction in various plant growth factors^59^. Prior treatment with melatonin notably enhanced Relative Leaf Water Content (RLWC) in rice plants under both salt and drought stress conditions (Fig. 3C). These outcomes are consistent with earlier findings from Ref.^60^, the observed rise in RLWC could be attributed to melatonin’s potential involvement in modulating stomatal behavior, effectively regulating their opening and closure to prevent undue water loss from leaves^61^. Electrolyte Leakage (EL) serves as an indicator of alterations in cell membrane structure during high salt and water deficit conditions. Our results show a notable increment in electrolyte leakage during salt and drought stress (Fig. 3B). Utilizing its relative conductivity allows for the assessment of damage to both the structure and function of cell membranes under various stresses^62^. Melatonin pre-treatment significantly decreased electrolyte leakage during salt and drought stress in rice plants. Similar results were obtained by Ref.^63^ in drought and Ref.^64^ in salt stress conditions. Consequently, the decrease in electrolyte leakage may be associated with elevated levels of CAT (catalase) and GSH (glutathione) by melatonin treatment during salt and drought stress conditions. This indicates that the utilization of melatonin might mitigate oxidative harm induced by salinity and drought stress.

Both salt and drought stress in rice plants trigger the excessive production of reactive oxygen species (ROS), which then leads to damage within various biomolecules. This disruption in the equilibrium between ROS generation and elimination adds to the overall oxidative stress within the plant’s system^65^. Melatonin is believed to act as an antioxidant in plants, aiding in cellular redox regulation, scavenging reactive oxygen species (ROS), and stabilizing plant cell membranes, thus offering protection against various environmental stressors^27,66^. Our results show that melatonin pre-treatment in rice suppressed the accumulation of ROS during salt and drought stress (Fig. 4A,B). These findings align with previous observations indicating that melatonin reduces ROS accumulation in watermelon and cucumber subjected to salt stress^67,68^ in maize and soybean subjected to drought stress^40,57^ with respect to non-treated plants.

Moreover, Li et al.^69^ reported that the application of exogenous melatonin enhanced plants’ tolerance to cold, drought, and salt stress. This effect was attributed to a reduction in reactive oxygen species (ROS) burst, maintenance of photosynthetic efficiency, decrease in malondialdehyde (MDA) levels, and enhancement of antioxidant activity in tea plants. Melatonin may have the capacity to enhance cellular redox homeostasis by stimulating the entire antioxidant system, encompassing both antioxidant enzymes (e.g., catalase, superoxide dismutase, peroxidase, ascorbate peroxidase, and monodehydroascorbate reductase) and non-enzymatic antioxidants (such as glutathione and ascorbate)^70^, as well as elevating levels of polyphenols^71^, carotenoids^72^, and anthocyanins^73^, to protect plants from abiotic stress-induced oxidative stress. Nonetheless, the precise mechanisms underlying this stimulatory action remain unclear. It is yet to be determined whether melatonin’s effect results from a direct interaction with existing enzymes or if it involves signal transduction mechanisms that regulate gene expression, leading to increased enzyme production.

During normal conditions, plants effectively neutralize reactive oxygen species (ROS) through both non-enzymatic and enzymatic antioxidants. However, under salt and drought conditions, the ROS production surpasses the capacity of the antioxidant defense systems, resulting in oxidative stress within the plant^74^. Catalase (CAT) and glutathione (GSH) are crucial antioxidants involved in vital processes within plant cells. Several studies on plants with altered levels of CAT and GSH proved the important roles of CAT and GSH in the tolerance of plants to environmental stresses^75^. The results of our study show that salt and drought stress slightly increase the level of CAT as shown in the (Fig. 4D). This slight increase in CAT activity may be due to its activation to encounter the accumulation of H_2_O_2_ induced by water shortage and salinity stress^76^. The findings from this study align with previous research indicating that plants respond to oxidative damage induced by various stressors by deploying mechanisms to maintain cellular equilibrium and withstand abiotic stress^44,77^. When the levels of glutathione within cells become more oxidized or decrease due to environmental factors, it triggers a signaling process, which prompts cells to react as though their glutathione levels are persistently low, assisting in their adaptation to changes in the environment^78^. The metabolism of glutathione (GSH) and the maintenance of the GSH pool are integral to plant responses to various abiotic stresses^79^. Several studies have highlighted a reduction in glutathione (GSH) levels in various plant species under stress conditions like salinity, extreme temperatures, and heavy metal exposure^80–82^. The study’s findings indicate a decline in GSH levels during salt and drought stress, as depicted in (Fig. 4C). This trend might be attributed to the activation of NADPH oxidase, which has a direct correlation with both ROS production and GSH levels^83^. However exogenous treatment of melatonin significantly increased the CAT and GSH activity during salt and drought stress (Fig. 4D). The results obtained in this study align with previous findings. Exogenous application of melatonin significantly boosted the activity of CAT in *Zea mays* L. and *Cynodon dactylon* L. when subjected to salinity stress^65,84^. Additionally, melatonin treatment has also shown an increase in CAT activity under various combined stresses like salinity and heat, drought, and cold stress^27,85,86^. In a plant cell, superoxide anion (O2^−^) can be rapidly converted to hydrogen peroxide (H_2_O_2_) by superoxide dismutase (SOD), while H_2_O_2_ can be scavenged by catalase (CAT)^87^, and melatonin’s involvement in boosting CAT activity contributes to maintaining the balance of reactive oxygen species (ROS) within the plant system. Similarly, melatonin plays a significant role in modulating glutathione (GSH) activity during abiotic stress conditions. Melatonin application appears to elevate the levels of Glutathione (GSH) in different plants after exposure to salt^67^, drought^86^, and heat stress^85^. Our study’s findings align with these results, as depicted in (Fig. 4C), which underscores the role of melatonin in enhancing GSH activity during stress conditions. The elevated GSH levels attributed to melatonin may indicate its role in modulating the AsA-GSH cycle, which plays a crucial role to detoxify the H_2_O_2_ and provide protection to plants from environmental stresses^88^.

Further we studied *OsSOS, NHX, HSF* and *DREB’*s expression level under salt and drought stress with different time points in response to melatonin. These genes are extensively studied and regarded as controllers of drought and salt stress response in rice plants. Different concentration of salt influence gene expression in rice, such as *SOS2*, and *NHX* were over expressed and directly activated the expression of K^+^/Na^+^ transporters, and regulate salt tolerance^89^. The expression of *OsSOS1* and *OsNHX1* was up-regulated in rice seedling by applying 100 mM of NaCl^90^. Overexpression of *OsSOS* under 150 mM of NaCl treatment showed improvement in growth parameters and retain relative water contents^32^. *OsNHX* genes play a crucial role in regulating the sodium (Na^+^) and potassium (K^+^) concentrations within the rice cytoplasm, aiding in the plant’s ability to manage and withstand salinity stress^91^. The *OsNHX* family genes exhibit regulation in salinity stressed rice plants, and the overexpression of *OsNHX1* imparts resistance to salinity stress in transgenic rice^33^. In accordance with Cattarin et al.^92^, who noted an increase in *OsNHX1* expression in the leaves of Pokkali and IR29 rice seedlings under 200 mM salinity stress, our study observed up-regulation of OsSOS and OsNHX in plants subjected to both salt stress and combined salt and drought stress (Fig. 5A,B). Exogenous melatonin improved salt tolerance by up-regulating the expression of *SOS* pathway in *Malus hupehensis* and *SOS1*, *SOS2*, and *SOS3* genes under salinity stress in Chinese medicinal herbs^93,94^. Melatonin treated plants showed over expression of ion transport proteins *NHX1* and *AKT1* during exposure to salt stress^95^. Consistent with previous findings, this study affirms that the external application of melatonin significantly contributes to alleviating salt stress by modulating the expression of *SOS* and *NHX* genes (Fig. 5A,B). Pretreatment with melatonin has been shown to boost the transcription of *OsSOS* and *OsNHX* in rice plants during salt stress, aiding in the removal of Na^+^ and maintaining plant resistance^95^.

Studies show that different transcription factors were identified which play an important role in the regulation of plants responses to different stresses^96^. In rice cultivar AP2 transcription activators *OsDREB1A* is up-regulated during drought and high salt stress^97^. *OsDREB1B* and *DREB1A* were up-regulated in *Arabidopsis* to enhanced dehydration and high salinity^98^. Over expression of *HSFA1* and *HSFA2* genes have been reported in soyabean, tomato and *Arabidopsis* to improve plant heat resistance^99,100^. A recent study showed that *OsHSFC1b* is overexpressed to improve salt tolerance in rice plants^101^. Moreover Scharf et al.^38^ confirmed that *HSFA3* is a part of drought stress signaling. The results of our study demonstrated that *OsHSF* and *DREB* are up-regulated in individual drought stress and drought, salinity combined stress (Fig. 5C,D), that show that *OsHSF* and *DREB* are the important transcriptional regulator during drought stress in rice plants. Exogenous treatment of melatonin enhanced carbohydrate metabolism and up-regulated transcription factors such as *DREB*, *HSF*, *WRKY*, and *MYB* in different plants^102^. Melatonin regulates various transcription factors like *DREB* in cotton^103^, and *DREB2A* in *Arabidopsis* under salinity and drought stress conditions^103^. In our study, melatonin treatment significantly elevated the expression of *OsHSF* and *DREB* genes in rice plants subjected to salt, drought stress individually, and their combined stress conditions (Fig. 5C,D). To summarize, there is a hypothesis that melatonin enhances rice plants’ response to salt and drought stress. In summary, it is hypothesized that melatonin improves the response of rice plants to salt and drought stress. Melatonin encounters the production of ROS within cells. As ROS levels rise, melatonin acts as an antioxidant, scavenging these ROS and boosting antioxidant activities. Melatonin seems to induce or activate the expression of specific resistance genes, thereby enhancing the plant’s ability to tolerate salt and drought stress. Further investigation is needed to comprehensively understand the mechanisms and signaling pathways involved in melatonin’s response under salt and drought stress in rice plants.

## Conclusion

Our study showed that both the salt and drought stresses induced oxidative damage by generation of ROS and membrane damage due to lipid peroxidation, which leads to reduction in rice plant growth and development. Exogenous melatonin application reduces salt, drought stress individually as well as in combine. Melatonin increased fresh and dry weight of rice under salt and drought stress. Similarly, melatonin treatment significantly reduced the accumulation of ROS and increased the antioxidant activity. Moreover, melatonin up-regulated the genes expression that are responsible for ion homeostasis. Future perspectives entail unraveling melatonin’s precise mechanisms, optimizing its application strategies, and validating its effectiveness in field trials for sustainable crop resilience under salt and drought stresses.

### Statement of adherence of the study to IUCN guidelines

The current study complies with relevant guidelines of IUCN Policy Statement on Research Involving Species at Risk of Extinction and Convention on the Trade in Endangered Species of Wild Fauna and Flora.

## Data Availability

The data presented in this study are available on request from the corresponding author.
